# Plant Protease Inhibitors in Therapeutics-Focus on Cancer Therapy

**DOI:** 10.3389/fphar.2016.00470

**Published:** 2016-12-08

**Authors:** Sandhya Srikanth, Zhong Chen

**Affiliations:** Natural Sciences and Science Education, National Institute of Education, Nanyang Technological UniversitySingapore, Singapore

**Keywords:** BBI, cancer, food crops, plant protease inhibitors, pharmacological, therapeutics

## Abstract

Plants are known to have many secondary metabolites and phytochemical compounds which are highly explored at biochemical and molecular genetics level and exploited enormously in the human health care sector. However, there are other less explored small molecular weight proteins, which inhibit proteases/proteinases. Plants are good sources of protease inhibitors (PIs) which protect them against diseases, insects, pests, and herbivores. In the past, proteinaceous PIs were considered primarily as protein-degrading enzymes. Nevertheless, this view has significantly changed and PIs are now treated as very important signaling molecules in many biological activities such as inflammation, apoptosis, blood clotting and hormone processing. In recent years, PIs have been examined extensively as therapeutic agents, primarily to deal with various human cancers. Interestingly, many plant-based PIs are also found to be effective against cardiovascular diseases, osteoporosis, inflammatory diseases and neurological disorders. Several plant PIs are under further evaluation in *in vitro* clinical trials. Among all types of PIs, Bowman-Birk inhibitors (BBI) have been studied extensively in the treatment of many diseases, especially in the field of cancer prevention. So far, crops such as beans, potatoes, barley, squash, millet, wheat, buckwheat, groundnut, chickpea, pigeonpea, corn, and pineapple have been identified as good sources of PIs. The PI content of such foods has a significant influence on human health disorders, particularly in the regions where people mostly depend on these kind of foods. These natural PIs vary in concentration, protease specificity, heat stability, and sometimes several PIs may be present in the same species or tissue. However, it is important to carry out individual studies to identify the potential effects of each PI on human health. PIs in plants make them incredible sources to determine novel PIs with specific pharmacological and therapeutic effects due to their peculiarity and superabundance.

## Introduction

Protease inhibitors (PIs) are well known as one of the prime candidates to have numerous applications in biotechnology and medicine. These are very important tools for better interpretation of basic principles of protein interactions and the designing of new compounds for the control of pathologic processes and many diseases (Lingaraju and Gowda, 2008). More than half of the world's population still depends completely on plants and their medicinal products. Until now, plants have served as starting raw materials for numerous drugs on the market. From ancient time, plant extracts containing proteolytic enzymes have been used in traditional medicine (De Feo, 1992). In Ayurvedic, Homeopathy, Unani and even in Allopathic systems, medical practitioners, and producers suggest and use several medicines that are prepared from whole plants or plant parts or phytochemicals such as secondary metabolites. Unlike primary and secondary metabolites which comprise of an array of several thousands of compounds, there are other compounds which are still less explored among plant natural products, particularly the proteins of low molecular weight. Protease inhibitors are among the most important groups of such proteins. Similar to animals and micro organisms, plants also contain several different types of protease inhibitors.

It has been reported that the occurrence rates of breast, colon, and prostate cancer are low in a population consuming a higher amount of seeds like beans, maize and rice (Correa, 1981). There are many lines of evidence from *in vitro, in vivo*, epidemiological and clinical trial data that demonstrate a plant-based diet decreasing the risk of chronic diseases, cancer in particular (Hasler, 1998). Block (1992) reported a review of 200 epidemiological studies which indicated that the cancer possibility in people who ate more fruits and vegetables was only one-half of that in those who ate a few of these foods. Thus, good nutrition is a recognized key to disease prevention. Also, health professionals are gradually recognizing the role of phytochemicals in disease prevention and health promotion. Recently, the molecular basis of disease prevention by nutritional involvement has been under scientific consideration whereby in the past, food intake has shown the ability to influence the initiation or progression of chronic diseases (Dirsch and Vollmar, 2001; Losso, 2008). Nowadays dietary factors have also attracted attention as cancer prevention agents due to their pharmacological safety. The abundance of plants can be used either as raw materials “as it is” or processed further to generate concentrated bioactive components with therapeutic action like flavonoids and phenolic acids that comprise powerful anti-oxidant activities (Pietta, 2000; Lizcano et al., 2010).

Food intake shows a strong influence on health (Losso, 2008). Instead of pills, functional foods can be consumed as part of a normal diet. Hence, plant protease inhibitors (PPIs) fit the definition of a functional food. Many researchers have classified these plant protease inhibitors into families such as Bowman-Birk, Kunitz, Potato I, Potato II, Serpine, Cereal, Rapeseed, Mustard, and Squash (Laskowski and Qasim, 2000; De Leo et al., 2002). Naturally occurring PIs are abundant in legume seeds. Serine protease Bowman-Birk inhibitors (BBI) and Kunitz-type inhibitors (KTI) have been studied extensively as compared to the other in these legumes (Ryan, 1990). These families differ in their mass, cysteine content, and the number of reactive sites (Lingaraju and Gowda, 2008). BBIs, which molecular weights range from 8000–10000 Da, are double-headed serine protease inhibitors with 71 amino acids and 7 disulphide bonds (Odani and Ikenaka, 1973). Each BBI has two functional active sites on opposite sides of the molecule, which inhibits both trypsin and chymotrypsin-like proteases (Clemente et al., 2011). Whereas KTIs are 8000–22,000 Da proteins with two disulphide linkages and a single reactive site of trypsin (Laskowski and Qasim, 2000). Among all, BBI is identified as a suitable protein to work with, in terms of handling, resistance to temperature extremities and acidification (Fields et al., 2012). Naturally occurring plant based BBI is considered as a therapeutically significant candidate in the treatment of multiple diseases, especially in the field of cancer prevention (Fields et al., 2012). Perhaps its greatest potential in therapeutic property lies in its ability to suppress carcinogenesis irreversibly even during early stage (Wattenberg, 1992). Encouragingly, the amount of BBI that reaches the internal organs after oral ingestion reaches the range that prevents malignant transformation *in vitro* (Yavelow et al., 1985; Fields et al., 2012). Several PPIs are under further evaluation in human clinical trials.

Protease inhibitors designed for therapeutic applications are quickly advancing due to the ever extending establishment of key information provided by the protein chemists and enzymologists working in this field. In this review, we focus on the role of plant proteases and their inhibitors in human diseases, and on the possible application of proteinaceous plant PIs as drugs. We also will discuss the several criteria to be met before such drugs are applicable to clinical trials.

## Roles of plant protease inhibitors in health and disease control

The widespread distribution of protease inhibitors throughout the plant kingdom is well known since 1938 (Ryan, 1973). In general, these PIs comprises about 5–10% of the total content of water-soluble proteins found in the seeds of dicots and monocots of angiosperms and in gymnosperms (Mutlu and Gal, 1999). However, the most well-studied protease inhibitors of plant origin are from three main families namely, Fabaceae, Poaceae, and Solanaceae (Richardson, 1991). Weder (1981) reported that the seed protein of the legumes enriched with up to 6% of PIs, whereas cereal contains about 10% of PIs (Pusztai, 1972). Later, many studies have reported PIs found in other families such as Malvaceae, Rutaceae, Poaceae and Moringaceae (Bijina et al., 2011). These natural PIs mainly accumulate in tubers, seeds, and leaves.

Medicinal plant biotechnology has emerged as a revolutionary methodology which is useful to induce the formation and accumulation of desirable compounds and eventually develop the therapeutic product (Constabel, 1990). Therefore, it is indispensable to select locally available edible plant species or plant extracts that could practically be added to the available drugs list, or even replace some expensive compounds that need to be utilized in pharmaceutical preparations. The investigation to search for PIs to combat several clinical disorders started in early 1950's (Vogel et al., 1968). For many years, several researchers have isolated and purified these plant PIs from different plant species and examined them as therapeutic agents using *in vitro* methods. Many of those naturally found PIs were further characterized from different plant species which mainly included trypsin from serine protease group which have been tested for various diseases (Richardson, 1991; Tamir et al., 1996; Majumdar, 2013). This review explains about PIs of all earlier reported plant species that have been used as therapeutic agents and tested against different diseases and human disorders (Table 1; Murugesan et al., 2001; Neuhof et al., 2003; Troncoso et al., 2003; Kobayashi et al., 2004; Lanza et al., 2004; Clemente et al., 2005, 2012; Kim et al., 2005; Suzuki et al., 2005; Capaldi et al., 2007; Banerjee et al., 2008; Tochi et al., 2008; Caccialupi et al., 2010; Hsieh et al., 2010; Joanitti et al., 2010; García-Gasca et al., 2012; Magee et al., 2012; de Paula et al., 2012a; Borodin et al., 2013; Ferreira et al., 2013; Rakashanda et al., 2013b; Clemente and Arques, 2014; Souza et al., 2014) and future scope to search for new species, which are as follows:

**Table 1:** **Pre-clinical (***in vitro***) studies and pharmacological effects of plant protease inhibitors (PPIs) in disease prevention**.

**Plant Name**	**Plant protease inhibitors**	**Model employed Cell/animal model**	**Concentration/Dose used**	**Effect observed**	**References**
*Bauhinia bauhinioides* (Mart.) J.F. Macbr. and *Bauhinia rufa* (Bong.) Steud.	BbCI, BrTI	Human prostate cancer cell lines DU145 and PC3	50–100 μM	Inhibited the cell viability of DU145 and PC3 cells caused an arrest of the PC3 cell cycle at the G0/G1 and G2/M phases	Ferreira et al., 2013
	BbCI, BrPI		BbCI (Ki = 5.3 nM), BrPI (Ki = 38 nM)	BbCI reduce edema formation	Neuhof et al., 2003
*Cicer arietinum* (Chickpea)	BBI	MDA-MB-231 (breast), PC-3 and LNCaP (prostate) lines	25–400 μg/ml	PIs inhibited the viability of MDAMB-231 breast cancer and PC-3 and LNCaP prostate cancer cells	Magee et al., 2012
*Coccina grandis* L. *Voigt*	14.3 kDa protease inhibitor	Colon cell lines	20–85%	Inhibits antifungal activity	Satheesh and Murugan, 2011
*Elusine coracana* (ragi)	RBI	K562 (leukemia)	5–40 μg/ml	RBI inhibits alpha-amylase and trypsin simultaneously	Sen and Dutta, 2012
*Enterolobium contortisiliquum*	EcTI	HCT-116 and HT29 (colorectal), SkBr-3 and MCF-7 (breast) K562 and THP-1 (leukemia), human primary fibroblasts, hMCs (human mesenchymal stem cells)	1.0–2.5 μM	Inhibit trypsin, chymotrypsin, plasma kallikrein, plasmin and inhibit activation of proMMP-9 and processing active MMP-2.	Nakahata et al., 2011
	EcTI	Gastric cancer cells	100–150 μM	Inhibit trypsin activity	de Paula et al., 2012a
*Fagopyrum sculentum* (buckwheat)	BWI-1, BWI-2a	JURKAT, CCRF-CEM (leukemia)		inhibitors have an anti-proliferative effect on T-acute lymphoblastic leukemia cells, like JURKAT, CCRF-CEM, and human normal blood lymphocytes	Park and Obha, 2004
*Glycine max* L. (Soybean)	Lunasin, BBI, KTI	NIH3T3 (Breast cancer mouse model)	360 ng/mg and 74.4 ng/mg protein	Lunasin is more effective than BBI	Hsieh et al., 2010
	BBI	MCF-7 (breast)	5–100 μM	BBI inhibited the proteasomal chymotrypsin-like activity in MCF-7 cells	Chen et al., 2005
	BBI, KTI	Sarcoma 37 cells, Human epidermoid carcinoma cells, Human cervical carcinoma cells	4–30 μg of BBI and 5–16 μg of KTI		Mark and Stephen, 1991
	BBI	HT29 (colon), CCD18-Co (colonic fibroblastic cells)	0.44–5.20 (IBB1) and 0.27–4.60 (IBBD2) mg/100 ml of soymilk	IBB2 inhibit trypsin-like proteases IBB1 inhibit trypsin or chymotrypsin-like proteases	Clemente and Arques, 2014
	SBTI	Red blood cells	61 ± 0.9 IU/mg	Inhibits trypsin	Borodin et al., 2013
*Hordeum vulgare* L. (Barley)	BSZx, BSZ4, BSZ7	Human plasma, leukocytes, pancreas	BSZ4–122 μg ml^−1^ BSZ7–381 μg ml^−1^ BSZx–133 μg ml^−1^	Inhibit trypsin and chymotrypsin	Dahl et al., 1996b
*Lavatera cashmeriana* Camb.	LC-pi I, II, III, and IV	THP-1 (leukemia), NCIH322 (lung), Colo205, HCT-116 (colon) lines PC3 (prostrate) and MCF-7 (breast) cancer cell lines		Inhibited trypsin, chymotrypsin and elastase proteases Inhibited cell proliferation and cell growth	Rakashanda et al., 2013a,b
*Macrotymola axillare* (E. Mey.) Verdc. (Perennial Horse gram)	BBI	Colorectal neoplasia	30 mg/kg	Inhibited lysosomal and proteasome-dependent proteolytic pathways	de Paula et al., 2012b
*Macrotyloma uniflorum* (Horse gram)	BBI	DMH (colorectal)	Fifteen lg total protein and 13 lM of fluorogenic substrates for a final volume of 240 lL, in 50 mM Tris–HCl pH 8.0 containing 10 mM MgCl2	A significant increase in the trypsin and chymotrypsin-like activities of the proteasome in preneoplastic lesions of the colon from DMH-treated animals	de Paula et al., 2012b
*Lens culinaris* L.	LCTI	HT29 (colon) CCD-18Co (colonic fibroblast) cells		Inhibited cell growth	Caccialupi et al., 2010
*Medicago scutellata* L.	MSTI	MCF7 (breast), HeLa (cervical carcinoma cells)	10 mg/ml	MSTI is an inhibitor of trypsin but no inhibitory activity toward chymotrypsin	Lanza et al., 2004
*Moringa oleifera* Lam.	Moringa protease inhibitor	Abdominal tumor	0.05, 0.1, and 0.2 mg/mL	Inhibits thrombin, elastase, chymotrypsin, trypsin, cathepsin and papain	Caceres et al., 1991; Cheenpracha et al., 2010; Mahajan and Mehta, 2010
*Peltophorum dubium* (Spreng.) Taub	Soybean kunitz type trypsin inhibitors	Human leukemia cells		Anti-proliferative effect on human leukemia cells (JURKAT) and induce apoptosis	Troncoso et al., 2007
*Phaseolus acutifolius* A. Gray (Tapery bean)	Pure TBPI and semi-pure Lectin fraction	Transformed murine fibroblasts and human cancer cell lines		Suppression of Matrix Metalloproteinase-9 activity	García-Gasca et al., 2012
*Pisum sativum* L. (Pea)	BBI (rTI1B and rTI2B)	Colon cancer cells		Inhibited growth of human colorectal adenocarcinoma HT29 cells *in vitro*	Clemente et al., 2005
	BBI	HT29 (colon) CCD18-Co (colonic fibroblast cells)	0–61 μM	rTI1B is active against trypsin and chymotrypsin.	Clemente et al., 2012
*Peltophorum dubium* (Spreng.) Taub.	PDTI	JURKAT (human leukemia cells) Nb2 rat lymphoma cells		PDTI triggered apoptosis	Troncoso et al., 2003
*Solanum tuberosum* L.	PT-1	Red blood cells, plant pathogenic microbial cells	0–20 mg/ml	Inhibited trypsin, chymotrypsin, and papain	Kim et al., 2005
	ST PIs		10–40 μg/ml	ST PIs has the highest inhibitory activity	
*Vicia faba* L. (Field bean)	FBPI	C6-glioma (tumor)	4.0 mg	Inhibited trypsin activity	Murugesan et al., 2001
	BBI	Mouse stomach carcinogenesis	1–2 mg of FBPI	Reductions in the multiplicity of gastric tumors	Fernandes and Banerji, 1995, 1996
*Vigna unguiculata* L. (Black-eyed pea)	BTCI	MCF-7 (breast)	2.0 μg/ml	BTCI is a more potent inhibitor for trypsin and against caspase-like and chymotrypsin-like	Souza et al., 2014
		MDA.MB.231 (highly invasive human breast cells), MCF-7 (human breast adenocarcinoma cells) and MCF-10A (normal mammary epithelial cells)	2.0–30.0 μg/ml	BTCI inhibited the cancer cell function directly by blocking the 20S proteasome core cavity	Mehdad et al., 2016

### *Ananas comosus* (L.) Merr. (pineapple)

Bromelain proteases from pineapple extracts (Fruit bromelain) are clinically used as anti-inflammatory agents (Ammon, 2002; Darshan and Doreswamy, 2004; Lemay et al., 2004) for colonic inflammation, chronic pain, rheumatoid arthritis, soft tissue injuries, and asthma (Izaka et al., 1972; Cooreman et al., 1976; Taussig and Batkin, 1988; Kelly, 1996; Maurer, 2001; Jaber, 2002; Hale et al., 2005). Similar folding and disulfide bond connectivity were found to be shared in Bromelain inhibitor VI from pineapple stem (Stem bromelain, BI-VI) with the Bowman-Birk trypsin/chymotrypsin inhibitor from soybean (BBI-I) (Hatano et al., 1996). It is essentially a group of sulfhydryl proteolytic enzymes that includes a variety of cysteine proteases (Tochi et al., 2008). Along with protease inhibitors, it also encompasses peroxidases, acid phosphatase, organically bound calcium and remains stable over a wide range of pH 2–9 (Hatano et al., 1996; Tochi et al., 2008). Even though, both stem bromelain and fruit bromelain are single-chain glycosylated enzymes, stem bromelain has low proteolytic ability and specificity for peptide bonds compared to fruit bromelain. Similarly, the molecular weight ranges for stem bromelain and fruit bromelain are 26–37 kDa and 24.5–32 kDa respectively (Grzonka et al., 2007; Gautam et al., 2010; Kumar et al., 2011).

It is reported that bromelain with its proteolytic nature is well absorbed orally in a dose-dependent manner (Hatano et al., 1996). Proteolytic activity of bromelain has been shown to play a minor part in pharmacological activity while other factors of it, such as immune-modulatory, hormone-like properties, fibrinolytic activity and uncharacterized components such as CCS and CCZ compliments also contributes toward its pharmacological activity (Tochi et al., 2008). However, it is necessary to investigate further on these uncharacterized components. Investigating how the uncharacterized components can be incorporated into daily food is important to determine if it is safe and non-toxic (Tochi et al., 2008).

### *Bauhinia bauhinioides* (Mart.) J.F. Macbr. and *Bauhinia rufa* (Bong.) Steud.

*Bauhinia* seeds are rich in serine and cysteine proteases inhibitors (Oliva and Sampaio, 2008; Oliva et al., 2010, 2011). Oliva et al. (1999) isolated the *Bauhinia bauhinioides* kallikrein inhibitor (BbKI) from seeds of *B. bauhinioides*, which is a 18-kDa protein with a similar primary structure to that of other plant Kunitz-type inhibitors but is devoid of disulphide bridges. BbKI was able to inhibit plasma kallikrein, plasmin, bovine trypsin, bovine chymotrypsin, porcine pancreatic kallikrein, and murine plasma kallikrein (Oliva et al., 1999). Nakahata et al. (2006) isolated another inhibitor from *Bauhinia rufa* and named it as BrTI (*B. rufa* trypsin inhibitor) that can inhibit human plasma kallikrein (K_i app_ = 14 nM) in addition to trypsin (K_i app_ = 2.9 nM).

Kunitz-type protease inhibitor from *B. bauhinioides and B. rufa* are reported to reduce the edema formation in isolated perfused rabbit lung (Neuhof et al., 2003). Kunitz-type Inhibitor BbCI (10(-5) M) from *B. bauhinioides* significantly decreased the pulmonary edema in isolated perfused rabbit lungs caused by activated neutrophils via release of elastase to the same degree as by eglin C (10(-5) M) from *Hirudo medicinalis*, which was used as a reference (Neuhof et al., 2003). Kallikreins play an important role in the establishment of a common prostate cancer. RGD/RGE motifs of the inhibitor BrTI from *Bauhinia rufa* is included into rBbKIm to form the recombinant *B. bauhinioides* kallikreins (Ferreira et al., 2013). This rBbKIm inhibited trypsin (K_i app_ = 1.6 nM), chymotrypsin (K_i app_ = 7.4 nM), and human plasma kallikrein (K_i app_ = 3.6 nM). Also, it was reported that the viability of fibroblasts was not affected when rBbKIm inhibited the cell viability of DU145 and PC3 prostate cancer cells (Ferreira et al., 2013).

### *Cicer arietinum* L. (chickpea)

Protease inhibitor concentrates extracted from Chickpea seeds which enriched in BBI-type PIs exhibited inhibitory activity against chymotrypsin (Magee et al., 2012). Viability of MDAMB-231 breast cancer and PC-3 and LNCaP prostate cancer cells were inhibited significantly by Chickpea Bowman-Birk-type protease inhibitor (molecular weight ~8 kDa) at all concentrations tested (25–400 μg/ml; Magee et al., 2012). The study also suggested that chickpea PIs may contain same anticancer properties like soybean BBI and hence deserves further study as a potential chemopreventive agent (Magee et al., 2012).

### *Coccinia grandis* (L.) Voigt.

The *Coccinia grandis* protease inhibitors (CGPI) is a protein of 14.3 kDa isolated from its leaves. CGPI showed a highest inhibitory activity against both bovine pancreatic trypsin and chymotrypsin. The antimicrobial activity of thses CGPIs has been reported by Satheesh and Murugan (2011). CGPIs exhibited significant growth inhibitory effect on colon cell lines via dose-dependent manner. These CGPIs also inhibited mycelial growth and sporulation of pathogenic microbial strains such as *Staphylococcus aureus, Klebsiella pneumonia, Escherichia coli, Proteus vulgaris, Bacillus subtilis* and pathogenic fungus *Mucorindicus, Candida albicans, Pencilliumnotatum, Aspergillus flavus*, and *Cryptococcus neoformans* (Satheesh and Murugan, 2011). PIs-treated fungus showed a significant shrinkage of hyphal tips. These results indicate that the PIs extracted from *C. grandis* will be an excellent compound to develop oral or other anti-infective agents.

### *Elusine coracana* Gaertn. (ragi)

Generally in many regions, finger millet or ragi is considered as a staple food crop for its excellent nutritional qualities, long storage capability, and medicinal properties. Sen and Dutta (2012) reported that purified ragi (*Elusine coracana*) is a 14 kDa bifunctional inhibitor (RBI), a member of cereal trypsin/α-amylase inhibitor family that simultaneously inhibits α-amylase and trypsin forming a ternary complex (Maskos et al., 1996). Its primary structure is a monomeric protein of 122 amino acids containing five intramolecular disulfide bonds (Campos and Richardson, 1983). The RBI reduced cellular proliferation and induced apoptosis of chronic myeloid leukemia cell, K562. These purified RBI exhibited cytotoxicity toward K562 chronic myeloid leukemia cells, but not against normal human peripheral blood mononuclear cells. This investigation may provide a future preventive as well as a natural therapeutic solution for chronic myeloid leukemia.

### *Enterolobium contortisiliquum* (Vell.) morong

It is a flowering tree in the pea family, Fabaceae. The purified *Enterolobium contortisiliquum* Trypsin Inhibitor (EcTI) is a 20 kDa protein of a Kunitz-type inhibitor from the seeds. EcTI inhibited the activity of trypsin, chymotrypsin, plasma kallikrein, and plasmin (Nakahata et al., 2011). The effects of a EcTI on adhesion, migration, and invasion of gastric cancer cells were reported by de Paula et al. (2012a). EcTI was shown to reduce the expression without affecting the fibroblasts upon adhesion and interrupt the cellular organization of molecules such as integrin β1, cortactin, MT1-MMP, MMP-2, and N-WASP, which are involved in the establishment and maturation of invadopodia. EcTI decreased Src-FAK signaling thereby inhibited the adhesion, migration and cell invasion (de Paula et al., 2012b). It was clear from the study that EcTI inhibited the invasion of gastric cancer cells by manipulating the integrin-dependent cell signaling pathways. Furthermore, Nakahata et al. (2011) reported the inhibition of human cancer cell lines, such as HCT116 and HT29 (colorectal), K562 and THP-1 (leukemia), SkBr-3 and MCF-7 (breast), as well as on human primary fibroblasts and human mesenchymal stem cells (hMSCs) upon treatment with EcTIs. Data indicates that the EcTI is an important tool in the studies of tumor cell development and distribution as it prevents proMMP activation, and is cytotoxic against tumor cells without disturbing normal tissue.

### *Fagopyrum sculentum* moench (buckwheat)

A buckwheat inhibitor (BWI)-1 protein extracted from common buckwheat seeds with a molecular weight of 7.7 kDa (Dunaevsky et al., 1997). BWI-1 is from the potato inhibitor I family which can inhibit trypsin, chymotrypsin, and subtilisin, whereas BWI-2a is a new PI, homologous to the vicilin family that can only inhibit trypsin (Lim, 2013). The inhibitory activity of BWI-1 and BWI-2a against T-acute lymphoblastic leukemia (T-ALL) cells, such as JURKAT and CCRF-CEM and human normal blood lymphocytes has been reported previously (Park and Obha, 2004; Lim, 2013). These two inhibitors induce apoptosis in these cells with DNA fragmentation.

### *Glycine max* (L.) Merr. (soybean)

**Figure 1:**
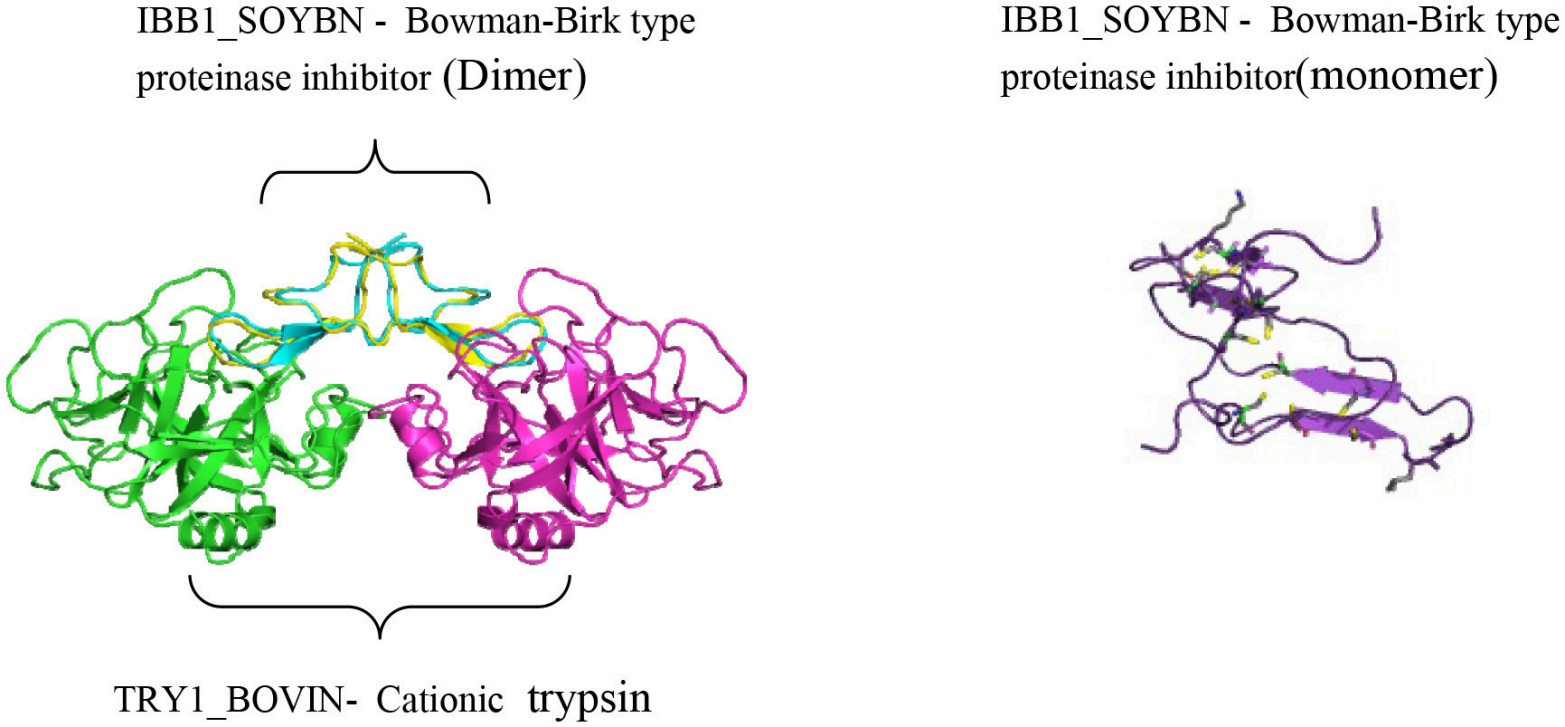
**Crystal structure of cancer chemopreventive Bowman-Birk inhibitor from Soybean in a ternary complex with bovine trypsin (PDB id: 1d16r) (Koepke et al., 2000)**.

The seeds of *Glycine max* possess both BBI and KTI. Its BBI is an 8 kDa protein and is able to reduce the proteolytic activities of trypsin, chymotrypsin, elastase, cathepsin G, and chymase, serine protease-dependent matrix metalloproteinases, urokinase protein activator, mitogen activated protein kinase, and PI3 kinase, and upregulates connexin 43 (Cx43) (Losso, 2008). Whereas the molecular weight of KTI is approximately 22 kDa. A concentrated protein extract of soybean, known as Bowman-Birk inhibitors concentrate (BBIC) which is enriched with BBI, is a present investigational new drug. The name of the family Bowman-Birk inhibitors is coined after D. E. Bowman and Y. Birk, who identified and characterized the typical member of this family, the soybean inhibitor from soybean (*Glycine max*; Bowman, 1946; Birk, 1961). In rodents, soybean BBI treatment has a potent suppressive effect on colon and anal gland inflammation, following exposure to carcinogenic agents (Billings et al., 1990). Soybean BBI reduced the initiation and regularity of colorectal tumors at low concentrations of 10 mg/100 g diet in the dimethylhydrazine (DMH) rat model, without any adverse effect on the animal growth or organ physiology (Kennedy et al., 2002). This suppressive effect on the colorectal tumor development was disappeared upon the reduction of BBI inhibitory activity, indicating that the BBI inhibitory activity against serine proteases may be necessary for the chemopreventive properties (Figure 1). The protease activities, which plays a major role in tumorigenesis, are deregulated in colorectal cancer and neoplastic polyps (Chan et al., 2010).

Ware et al. (1999) reported that soybean BBI inhibited the proteases of inflammation-mediating cells and suppressed the superoxide anion radical production from the immunocytes. This study also indicated that BBIC can positively influence the dextran sulfate sodium-treated mice, which in turn useful in treating the human inflammatory bowel diseases, mainly ulcerative colitis. Another study reported that BBI is an anti-carcinogenic serine protease inhibitor that may obstruct the protease activity of prostate-specific antigen (PSA) and the development of human prostate cancer xenografts in nude mice (Wan et al., 1999b).

The role of soybean BBI in suppressing the development of activated oxygen species from prostate cancer cells (Sun et al., 2001), and in triggering DNA repair via a p53-dependent mechanism have been reported (Kennedy et al., 2002). In the recent studies, BBIC has prevented the growth of prostate tumors by its antiproliferative activity through stimulation of connexin 43 expressions in transgenic rats (McCormick et al., 2007; Tang et al., 2009).

The suppressive activity of soybean BBI, on HT29 colon cancer cells, compared with CCD-^18^Co cells of non-malignant colonic fibroblast was studied by Clemente et al. (2010). The study indicated that the BBI treatment in a dose-dependent manner, blocked the G0-G1 phase to disrupt the cell cycle distribution pattern of HT29 colon cancer cells. Chen et al. (2005) reported the BBI as a potential inhibitor which inactivated the MCF7 cancer cells both *in vitro* and *in vivo* by inhibiting 26S proteasomal chymotrypsin-like activity. The study also indicated that the BBI arrested the growth of MCF7 cells at G1/S phase, by accumulating MKP-1 causing interruption in the ubiquitin-proteasome pathway which resulted in suppressed ERK1/2 activity. Being a potential chemotherapeutic drug, BBI obstructs the cell proliferation and viability at different stages of cancer development. Hence, the BBI involving proteasome inhibition is considered as a new mechanism which may be a potential reason for its chemopreventive properties. Eventually, the study on soybean BBI proteins has provided an information on the therapeutic interference of soybean BBI for breast cancer treatment.

Kobayashi et al. (2004) studied the effects of soybean trypsin inhibitor (SBTI) on the enzymatic activity of extracellular uPA and signal transduction mechanisms that are involved in its expression and incursion into HRA human ovarian cancer cells. Authors concluded that KTI could stop cell invasiveness by reduction of uPA signaling instead of BBI although the mechanisms of KTI may not be the same as those of Bikunin. BBI inhibited M5067 ovarian sarcoma by increasing expression of tumor-suppressor molecules Cx43 (Suzuki et al., 2005).

Oral administration has been recognized as a possible and cost-effective approach to reducing cancer morbidity and mortality by inhibiting precancerous events before the occurrence of clinical disease (Prasain and Barnes, 2007). Hsieh et al. (2010) found that lunasin is actually the bioactive cancer preventive agent in BBIC and BBI simply protects lunasin from digestion when soybean and other seed food are eaten by a human. Currently, the rising of breast cancer cases urged to identify novel compounds that can be utilized as preventive or therapeutic agents. Evidence is shown from animal experiments, epidemiological studies, and human surveys of people consuming a soy-rich diet exhibited lower disease occurrence and mortality from breast cancer which leads to more research on various compounds from soy that can prevent breast cancer (Adlercreutz et al., 1995; Banerjee et al., 2008; Hernández-Ledesma et al., 2009). Consuming such anticancer compounds daily could be an alternative to chemotherapy, which is safe to the physiology of normal tissue and prevents initiation of micro tumors (Béliveau and Gingras, 2007; de Kok et al., 2008). Furthermore, it is important to assess the possible risks and advantages of phytochemicals to human health by understanding the physiological behavior of these compounds after oral intake which includes absorption, distribution, metabolism and excretion (Prasain and Barnes, 2007).

Borodin et al. (2013) reported that the effect of both proteins, soybean SBTI and aprotinin, on coagulation and thrombocyte hemostasis by *in silico* and *in vitro* methods and demonstrated the inhibition of blood clotting, fibrinolysis and platelet aggregation (**Figure 5**). These outcomes were accomplished by increased prothrombin time, activated partial thromboplastin time, activated clotting time, thrombin time as well as prevention of fibrinolysis.

### *Hordeum vulgare* L. (barley)

In 1970's, a group of ~43 kDa proteins from mature grains of barley (*Hordeum vulgare* L.) were identified as members of the serpin superfamily (Hejgaard et al., 1985). There are three mostly recognized barley serpin subfamilies: BSZ4, BSZ7, and BSZx. Barley serpins are effective, irreversible inhibitors of serine proteases of the chymotrypsin family. The Barley serpin, BSZx inhibits both chymotrypsin and trypsin at overlapping reactive sites (Dahl et al., 1996a). BSZx serpin interacts with a range of serine proteases from human plasma, leukocytes, pancreas, a fungal trypsin and three subtilisins (Dahl et al., 1996b). The study indicated that BSZx inhibitors which inhibited trypsin and chymotrypsin are also capable to inhibit coagulation factors such as thrombin, plasma kallikrein, Factor VIIa and Factor Xa.

### *Ipomoea batatas* L. (sweet potato)

The anti-proliferative effect and the mechanism of a 22 kDa trypsin inhibitor (TI) protein from sweet potato (Ipomoea *batatas* (L.)) storage roots on NB4 promyelocytic leukemia cells was reported by Huang et al. (2007). TI arrested cell cycle at the G1 phase as determined by flow cytometry analysis and apoptosis as shown by DNA laddering. TI-induced cell apoptosis involved p53, Bcl-2, Bax, and cytochrome c protein in NB4 cells. P53 and Bax proteins accumulated, and antiapoptotic molecule Bcl-2 decreased in the tested cells in a time-dependent manner during TI treatment. The study indicated that TI stimulates the apoptosis of NB4 cells by blocking the cell growth and activating the pathways of caspase-3 and -8 cascades.

### *Lavatera cashmeriana* Camb.

It belongs to the Malvaceae family, which is indigenous to Kashmir valley, has incredible medicinal values. For many years, its plant parts are being utilized to cure cold and sore throat (Rakashanda et al., 2013a). *Lavatera cashmeriana* protease inhibitors (LC-pi I, II, III, and IV) were extracted from its seeds and are considered as a Kunitz type of inhibitor based on their molecular size (Rakashanda et al., 2012, 2013a). These protease inhibitors were able to inhibit trypsin, chymotrypsin, and elastase. Four serine protease inhibitors appeared as 20.9, 14.1, 16.8, and 7.9 kDa peaks in gel filtration and 10, 14, 16, and 7 kDa bands by SDS PAGE. Representing that LC-pi I includes two similar subunits of 10 kDa. LC-pi I tested against *Klebsiella pneumoniae* and *Pseudomonas aeruginosa* which showed strong antibacterial activity but was less active against *Escherichia coli* (**Figure 5**). However, all four inhibitors demonstrated the *in vitro* anticancer activity on THP-1 (leukemia), NCIH322 (lung), and Colo205, HCT-116 (colon). Among all, LC-pi I and II were treated as potential anticancer agents (Rakashanda et al., 2013b). Moreover, a strong inhibitory effect of LC-pi I was exhibited in *in vitro* conditions on the initiation of the prostate (PC-3) and breast (MCF-7) cancer cells lines because of its protease inhibitor activity of trypsin, chymotrypsin, and elastase (Rakashanda et al., 2013a).

### *Lens culinaris* L. (lentil)

**Figure 2:**
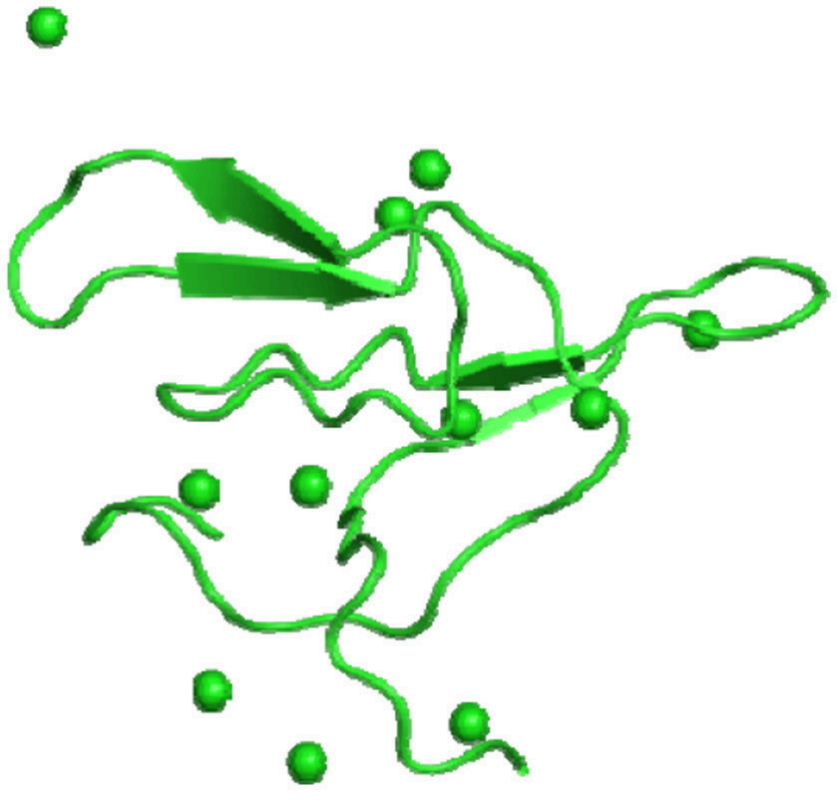
**IBB_LENCU—Bowman-Birk type protease inhibitor monomer from ***Lens culinaris*** (PDB id: 2aih)**.

Ragg et al. (2006) reported the isolation of a lentil (*Lens culinaris*, L., var. Macrosperma) seed trypsin inhibitor (LCTI) and its functional and structural characterization. LCTI is a 7.4 kDa double-headed trypsin/chymotrypsin inhibitor (Figure 2). Further, Caccialupi et al. (2010) isolated a full-length cDNA, encoding a BBI from lentil seeds. The effects of this mature BBI on the growth of human colon adenocarcinoma HT29 and colonic fibroblast CCD-^18^Co cells were evaluated. LCTI was able to inhibit the growth of such cells at concentrations as low as 19 μmol/L, in a concentration-dependent manner without affecting the CCD-^18^Co cells.

### *Macrotymola axillare* (E. Mey.) Verdc. (Perennial Horse gram)

Joubert et al. (1979) isolated and characterized two protease inhibitors, DE-3 and DE-4 from *Macrotyloma axillare* seeds. The amino acid sequences of two protease inhibitors were compared with the known sequences of the BBIs which resulted in 67% homology and corresponding properties of the BBI double-headed protease inhibitor group. Each of them comprise 76 amino acid residues including 14 half-cystine residues. BBI from the *M. axillare* worked against inflammation and initiation of pre-neoplastic lesions in the induced DMH mouse model as reported by de Paula et al. (2012b). In this study, the potential ability of BBI preparations was examined for the prevention of colorectal neoplasia caused by intraperitoneal injections of 1,2-dimethylhydrazine (DMH) by using 30 mg/kg dosage over a period of 12 weeks. Histopathological variations consistent with tumor development, increased CD44 expression and proteasome peptidase activities were exhibited by the DMH-treated mice. It is demonstrated that the BBI inhibited both lysosomal and proteasome-dependent proteolytic pathways through which it could prevent the development of pre-neoplastic lesions.

### *Medicago scutellata* L. (Snail medic)

**Figure 3:**
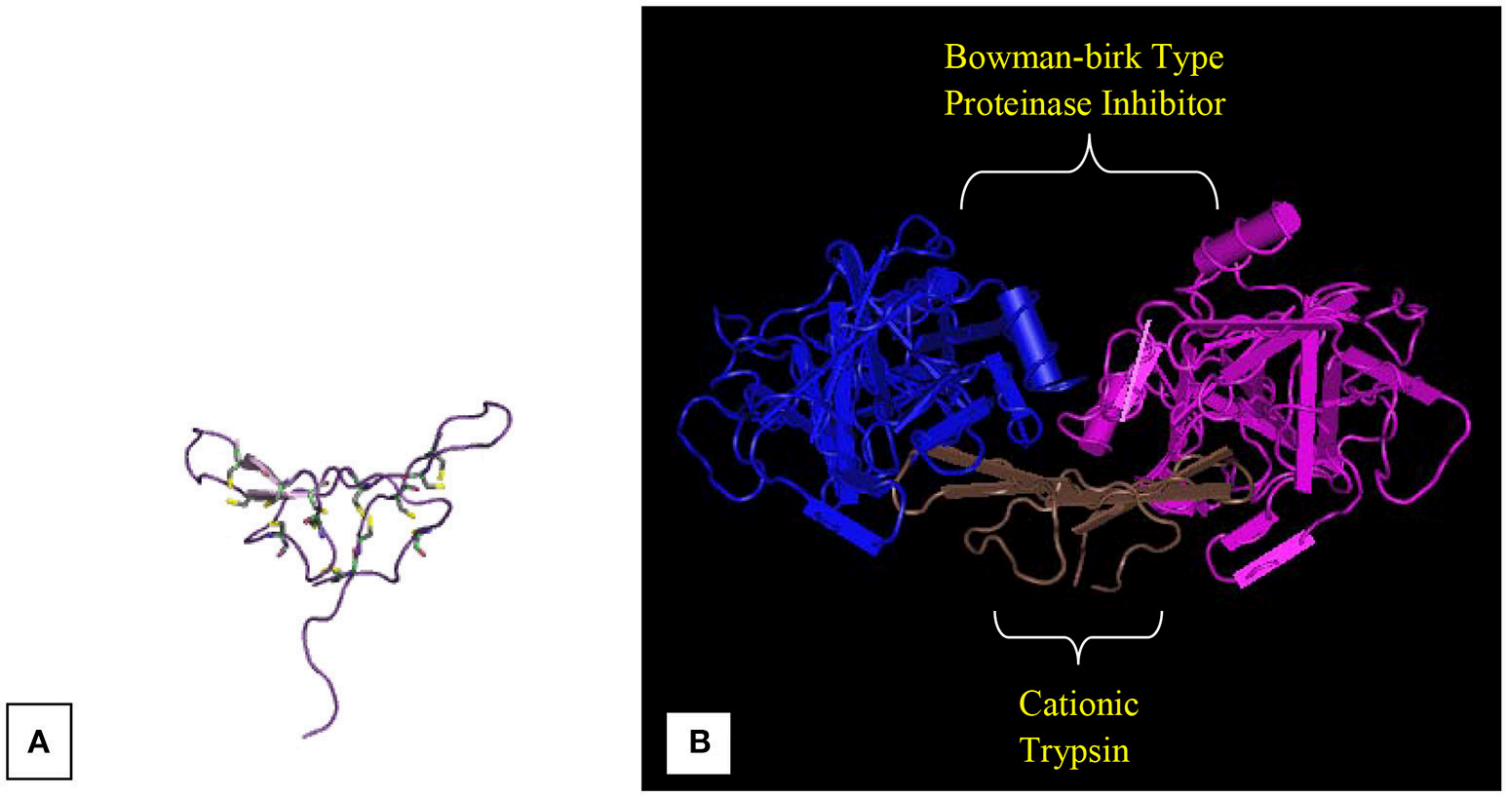
**Bowman-Birk inhibitors of ***Medicago scutellata*****. **(A)** IBB_MEDSC-Bowman-Birk type protease inhibitor (PDB id: 1mvz). **(B)** The anticarcinogenic Bowman-Birk inhibitor from snail medic (*Medicago scutellata*) seeds complexed with bovine trypsin (Capaldi et al., 2007).

*Medicago scutellata* L. (Snail medic) seeds stores a significantly higher content of a trypsin inhibitor (MsTI) compared to *Medicago* species. MsTI belongs to the BBI family of serine proteases and exhibited a highest sequence homology with the soybean BBI (Catalano et al., 2003). It consists of 62 residues corresponding to a molecular mass of 6.9 kDa. Catalano et al. (2003) reported the anticarcinogenic BBI, which is a high-resolution three-dimensional structure purified from snail medic seeds (*Medicago scutellata)* (MSTI) (Figure 3). Later, Capaldi et al. (2007) reported the ternary complex structure of the BBI that is purified from snail medic seeds (MSTI) and two molecules of bovine trypsin. The effects of MsTl on cell killing induced by cisplatin in MCF7 human breast carcinoma cells and HeLa cervical carcinoma cells were evaluated by Lanza et al. (2004). After 24 h of MsTI treatment with cell culture medium, resulted in increased cisplatin-induced cytotoxicity and reduced the clonogenic survival of MCF7 and HeLa cells in a dose-dependent manner. In comparison with the similar ternary complex of the SBTI, this model shows very little differences in the polypeptide chain of the trypsin binding sites. The area between Asp 26 and His 32 of the MSTI has the largest difference whereby the soybean inhibitor has an extra Leu inserted in position 29 (Catalano et al., 2007).

### *Moringa oleifera* Lam.

It belonging to the family Moringaceae, recorded high level of protease inhibitor activity (92%) against trypsin (Bijina et al., 2011). PI extracted from *M. oleifera* is a small protein with a molecular weight of 23.6 kDa. Its molecular mass and and the disulphide content of the polypeptide indicated that *Moringa* PI belongs to the Kunitz type of serine protease inhibitor family (Bijina et al., 2011). *M. oleifera* grows well throughout the tropics and almost every part of the plant is of value as food. The flowers, leaves, and roots are used in folk remedies for the treatment of tumors and the seeds for abdominal tumors. The bark is regarded as antiascorbic and exudes a reddish gum with properties of tragacanth is sometimes used for diarrhea. Roots are bitter and act as a tonic to the body and lungs. They are used as an expectorant, mild diuretic, stimulant in paralytic afflictions, in epilepsy, and in hysteria (Hartwell, 1971). Moreover, there are a few low molecular weight bioactive compounds present in *Moringa* seeds which exhibited bactericidal, fungicidal, immune suppressive activities (Mahajan and Mehta, 2010) and some anti-inflammatory agents (Caceres et al., 1991; Cheenpracha et al., 2010). Moringa PI is the potential source for the development of a new drug against thrombin, elastase, chymotrypsin, trypsin, cathepsin, and papain in pharmaceutical industries (Bijina et al., 2011). Furthermore, these PIs could also be used as a seafood preservative against proteolysis and in other biotechnological applications.

### *Phaseolus acutifolius* A. gray (Tepary bean) and *Phaseolus vulgaris* L.

Bowman-Birk PI from Tepary bean (TBPI) seeds with anti-trypsin and antichymotrypsin activities was isolated and characterized by Campos et al. (1997). García-Gasca et al. (2012) again purified and characterized 7 kDa TBPI as described by Campos et al. (1997). However, this purified TBPI did not show cytotoxicity but it was responsible for the increase in cell adhesion and decrease in extracellular matrix degradation in cell culture which leads to a decreased *in vitro* cell invasion capacity and suppression of matrix metalloproteinases-9 activity simultaneously. According to this study, the Tepary bean seeds contain minimum two separate groups of bioactive proteins with anticancer properties (García-Gasca et al., 2012). The study also reported that cells treated with TBPI diminished their invasive ability most probably due to the suppression of MMP2 and MMP9. Sun et al. (2010) isolated and purified two trypsin inhibitors of each with a molecular mass of 16 kDa from *Phaseolus vulgaris* cv “White Cloud Bean.” Both of these inhibitors showed an antiproliferative activity toward Leukemia L1210 albeit with a little variance in potency, but there was little activity toward lymphoma MBL2 cells.

### *Pisum sativum* L. (pea)

The crystal structure of the *Pisum sativum* (PsTI) isoform was determined by molecular replacement at 2.7A resolution using the X-ray coordinates of the soybean inhibitor as a search model (Li de la Sierra et al., 1999). Protease inhibitors, rTI1B, and rTI2B from *P. sativum* L. (Pea) are homologous to BBI with molecular mass range 7–9 kDa, but differ in inhibitory activity, on the growth of human colorectal adenocarcinoma HT29 cells *in vitro* (Clemente et al., 2005, 2012). The rTI1B proved to be active against trypsin and chymotrypsin, whereas the related mutant protein was inactive against both serine proteases. The proliferation of HT29 colon cancer cells was notably affected by rTI1B in a dose-dependent manner, whereas the inactive mutant did not show any significant activity on cell growth of colon cancer. Likewise, none of the recombinant proteins affected the growth of non-malignant colonic fibroblast CCD-^18^Co cells. From the findings, it was clear that serine proteases are important candidates in investigating the potential cancer preventive trait of BBI in the initial stages of colorectal cancer (Clemente et al., 2012; Clemente and Arques, 2014).

### *Peltophorum dubium* (Spreng.) Taub.

Troncoso et al. (2003) isolated a trypsin inhibitor, PDTI of molecular mass range 20–22 kDa from *Peltophorum dubium* seeds. The amino-terminal sequences of PDTI were the same as Kunitz-type soybean trypsin inhibitor (SBTI). Both PDTI and SBTI triggered apoptosis of Nb2 rat lymphoma cells via reducing viability, DNA fragmentation, DNA hypodiploidy and caspase-3-like activity. However, they did not damage normal mouse splenocytes or lymphocytes but caused apoptosis of concanvalin A-stimulated mouse lymphocytes (Troncoso et al., 2003). Many findings reported about the anti-proliferative effect and apoptosis in human leukemia cells (JURKAT) via PDTI and SBTI activity. In addition, human peripheral lymphocytes, stimulated with phytohemagglutinin or not, are also sensitive to viability decrease that is induced by SBTI (Troncoso et al., 2007).

### *Pseudostellaria heterophylla* Rupr. & Maxim. (Ginseng)

Wang and Ng (2006) isolated a 20.5 kDa trypsin inhibitor of Kunitz-type with antifungal activity (**Figure 5**) and a new lectin from roots of *P. heterophylla*. They exhibited a trypsin inhibitory activity that is similar to soybean trypsin inhibitor. Antifungal activity was also demonstrated toward *Fusarium oxysporum* like sprotinin and Kunitz-type trypsin inhibitors from soybeans and lima beans.

### *Solanum tuberosum* L.

Although protease inhibitors are found in plants belonging to different kinds of systematic groups, high levels of protease inhibitors are often reported in many plants belonging to the *Solanaceae* family (Plate et al., 1993). The effect of a protease inhibitor extracted from potatoes (POT II) which increase CCK release, on food intake was examined in 11 lean subjects by Hill et al. (1990). The findings suggested that endogenous CCK may be important in the control of food intake and that protease inhibition may have therapeutic potential for reducing food intake. Several antimicrobial peptides have been purified from potato tubers. As an example, a 5 kDa pseudothion of *S. tuberosum* (Pth-St1) was found to be active against bacterial and fungal pathogens of potato such as *Clavibacter michiganensis* subspecies *sepedonicus, Pseudomonas solanacearum*, and *Fusarium solani*. Trypsin or insect α-amylase activities are not inhibited by Pth-St1 and it does not affect cell-free protein synthesis or β-glucuronidase activity with true thionins (Moreno et al., 1994). It was also found that Snakin-1 (stSN1) and Snakin-2 (stSN2) are active against the fungal pathogens, *Clavibacter michiganensis* subspecies *sepedonicus* and *Botrytis cinerea* at concentrations <10 μM (Kim et al., 2009). Patatin, a potato tuber storage protein, which was purified to homogeneity, has antioxidant and antiradical activity (Liu et al., 2003). Potamin-1 (PT-1), a stress-inducible trypsin inhibitor, was also present in potato tubers (Ledoigt et al., 2006). The potamin-1 (PT-1) trypsin-chymotrypsin protease inhibitor (5.6 kDa) was found to be thermostable without potential hemolytic activity but holds antimicrobial activity. It strongly inactivated the pathogenic microbial strains such as *Candida albicans, Rhizoctonia solani*, and *Clavibacter michiganense* sub sp. michiganinse (Kim et al., 2005).

### *Vicia faba* L. (field bean)

A trypsin/chymotrypsin inhibitor with a molecular weight of 18 kDa was purified from seeds of *Vicia faba* L (Gupta et al., 2000). Fernandes and Banerji (1995) tested the ability of the FBPI, when administered by gavage, to subdue benzopyrene (BP)-induced neoplasia of the forestomach of mice. The mice that were treated with heat-inactivated FBPI showed similar tumor multiplicity to the BP-treated group, indicating that inhibitory capacity. These findings indicated the ability of FBPIs as effective chemo-protectors against gastric cancer in animals and, possibly, in humans as well. The purified FBPI has been found to be quite similar to the Soybean-derived BBI in its properties and anticarcinogenic potentials (Banerji and Fernandes, 1994; Fernandes and Banerji, 1995, 1996). Skin carcinogenesis can be effectively suppressed by topical treatment with a FBPI as reported by Fernandes and Banerji (1996) and plasmin inhibitory activity of FBPI can help to stop pulmonary metastasis of B16F10 melanoma cells systemically injected into BDF_1_ mice (Banerji et al., 1998a). Banerji et al. (1998b) investigated the ability of FBPI to stop ethylnittrosourea (ENU)-induced tumors of the nervous system of Sprague-Dawley rats. Murugesan et al. (2001) labeled the same FBPI with ^99m^${\mathrm{TcO}}_{4}^{-}$ to determine its capability to identify tumors in tumor-bearing rat models when a neural tumor incidence of 100% in the rats treated with heat-inactivated FBPI confirmed that the tumor suppressor activity of FBPI is related to its PI activity (Banerji et al., 1998b). This labeling was done with Sn^2+^ as a reducing agent and the yield was 95% which was stable for 2 h at ambient temperature. This study indicated that ^99m^TcFBPI has the exact prospective for imaging gliomas and probably other tumors as well. The study also indicated that it was possible to label FBPI with radionuclides such as ^186^Rh and ^153^Sm, which could also be used for the detection of tumors particularly of glial origin in patients.

### *Vigna unguiculata* L. (Black-eyed pea)

The Black-eyed pea Trypsin/Chymotrypsin Inhibitor (BTCI) isolated and purified from *Vigna unguiculata* (Cowpea) seeds is a natural PPI, and it belongs to the BBI family with two different and independent reactive sites for trypsin (Lys26) and chymotrypsin (Phe53) (Souza et al., 2014). BTCI is a globular protein comprising 83 amino acid residues with seven disulfide bonds and molecular weight of 9.1 kDa (Barbosa et al., 2007).

**Figure 4:**
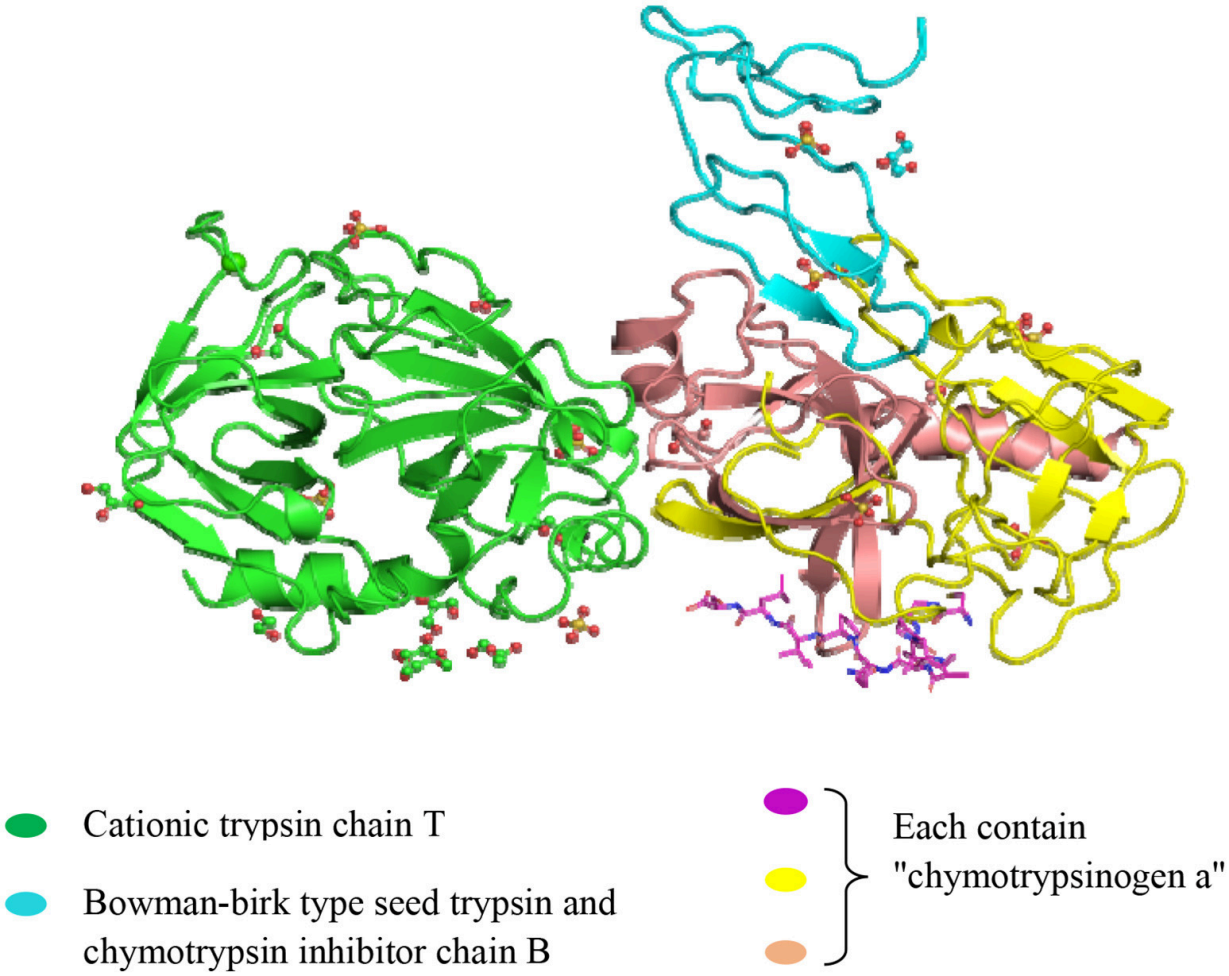
**Crystal structure of the Bowman-Birk serine protease inhibitor BTCI in complex with a copy of cationic trypsin and three copies of chymotrypsinogen A**. (http://www.ebi.ac.uk/pdbe-srv/view/entry/3ru4/summary.html).

The effects of the BTCI (Figure 4), on the MCF-7 breast cancer cells was reported by Joanitti et al. (2010). BTCI-induced apoptosis, cell death due to morphological alterations of the cell and lysosome membrane permeation demonstrated via cytostatic and cytotoxic findings. The anti-carcinogenic capabilities of BBI and newly identified BTCI established a promising tool for drug developments aimed to treat breast cancer. Recently, Souza et al. (2014) reported the effects of a BTCI on proteasome 20S in MCF-7 breast cancer cells and the catalytic activity of the purified 20S proteasome from horse erythrocytes, as well as the structural analysis of the BTCI-20S proteasome complex. Furthermore, Mehdad et al. (2016) stated that BTCI significantly decreased human breast adenocarcinoma cell viability by inhibiting the activity of proteasome 20S, with an associated cytostatic effect at the G2/M phase of the cell cycle and an increase in apoptosis.

Numerous protease inhibitors have been isolated and characterized from different plant species. PIs were isolated from *Scopolia japonica* cultured cells and these were found to have potential inhibitory activity against trypsin, chymotrypsin, kallikrein, plasmin, and pepsin but could not inhibit pain using synthetic or natural substrates (Sakato et al., 1975). Proteases like papain from *Carica papaya* L. has been used in different industrial processes including pharmaceutical process (Macalood et al., 2013). BBI like inhibitors were also isolated from sunflower (sunflower trypsin inhibitor-1; Luckett et al., 1999; Korsinczky et al., 2001; Marx et al., 2003; Lęgowska et al., 2010) and peanut (*Arachis hypogaea* L.; Tur Sinal et al., 1972; Norioka and Ikenaka, 1983; Suzuki et al., 1987). Protease inhibitors belonging to the Kunitz inhibitor family has been purified from pigeonpea (*Cajanus cajan* (L.)) PUSA 33 variety (Haq and Khan, 2003). A red gram protease inhibitor (RgPI) was extracted and purified from *Cajanus cajan* (Kollipara et al., 1994; Prasad et al., 2010b). BBI type inhibitors were purified and characterized from /in black gram (*Vigna mungo* (L.) Hepper) (Prasad et al., 2010a).

Mustard trypsin inhibitor was isolated and sequenced from the seeds of *Sinapis alba* L., members of the family *Cruciferae*. It was a serine protease inhibitor but did not show any structural similarity with other identified families of serine protease inhibitors from plants and it comprised more cysteine and glycine residues (Menegatti et al., 1985, 1992). In contrary, a similar type of PI was isolated and characterized from *Brassica napus* (rapeseed) (Ceciliani et al., 1994; Ascenzi et al., 1999). Inhibitors from *Secale cereale* L. and *Ricinus communis* L. also exhibited trypsin inhibitory activity (Odani et al., 1983). A few reports are available regarding the dual inhibitory activity (both serine proteases and α- amylase inhibitory activity) of some inhibitors from *Zea mays* L. (maize) (Filiz et al., 2014). Potato inhibitors II has been reported in flowers of *Nicotiana alata* Link & Otto (tobacco) (Atkinson et al., 1993). Serine protease inhibitors were reported in phloem sap of *Cucurbita maxima* (Kuroda et al., 2001), *Arabidopsis* and rice (*Oryza sativa* cv. Nipponbare; Fluhr et al., 2012) and pumpkin (*Cucurbita maxima*; Yoo et al., 2000). Squash Inhibitors have been reported in cucumber, zucchini (Wieczorek et al., 1985), watermelon (*Citrullus vulgaris)*, spaghetti squash, red bryony (*Bryonia dioica)*, figleaf gourd (Otlewski et al., 1987; Polanowski et al., 1987), *Momordica repens* (Joubert, 1984), wild cucumber (*Cyclanthera pedata*; Kuroda et al., 2001), wax gourd (*Benincasa hispida* (Thumb) cogn) (Atiwetin et al., 2006) and in several other plants of cucurbit family. The small size, structural rigidity and stability of the members of the squash inhibitor family serve as potential materials for studying serine protease-protein inhibitor interaction (Otlewski and Krowarsch, 1996). Cysteine protease inhibitors were reported in Rice (oryzacystatin) (Abe et al., 1987a,b), maize (Abe et al., 1992), apple fruit (Ryan et al., 1998), and several other monocotyledonous as well as dicotyledonous plants (Pernas et al., 1998; Sakuta et al., 2001). Aspartic protease inhibitor reported in *Anchusa strigosa* (Abuereish, 1998) and squash inhibit pepsin, which is a digestive aspartic protease.

## Naturally-occurring plant protease inhibitors in clinical trials

**Figure 5:**
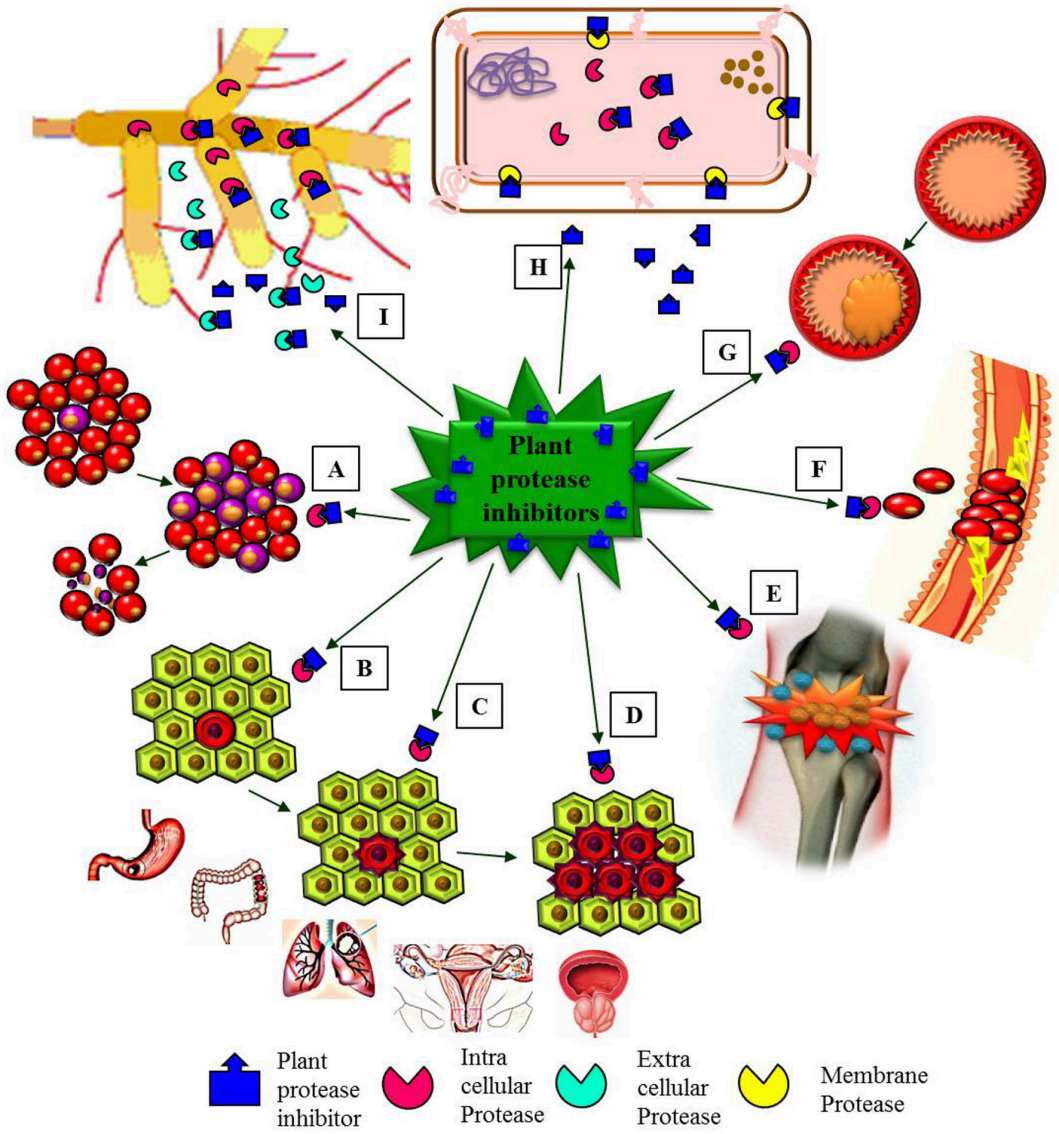
**Schematic representation emphasizing major pharmacological effects of plant protease inhibitors (PPIs)**. **(A)** PPIs induces apoptosis of leukemia cells (healthy red blood cells and leukemia blood cells). **(B–D)** PPIs showed *in vitro* anti-carcinogenic effects in initiation **(B)**, proliferation **(C)**, and progression **(D)** stages of different types of cancers (gastric, colon, lung, ovarian, and prostate cancers). **(E)** PPIs as anti-inflammatory agents. **(F)** The role of PPIs as anti-coagulant. **(G)** PPIs active against cardiovascular diseases (plaque formation in a blood vessel). **(H)** Antibacterial activity of PPIs. **(I)**. Antifungal activity of PPIs.

Many human clinical trials to assess the effect of BBIC have been finalized or are in progress (Armstrong et al., 2000b; Malkowicz et al., 2001; Lichtenstein et al., 2008). BBI clinical studies were carried out in patients (with the approval of Food and Drug Administration) with oral leukoplakia, prostrate cancer, gingivitis, ulcerative colitis, lung cancer, anti-inflammation (Figure 5), multiple sclerosis and encephalomyelitis using BBIC dose up to 1066 chymotrypsin inhibitory units (C.I.U) per day for single patient (Sugano, 2006; Table 2). During human trials, even though the BBI level could not be found in the blood after oral BBIC medicating, it could be detected in urine (Wan et al., 2000).

**Table 2:** **Soybean Bowman-Birk inhibitor (BBI) in clinical studies**.

**Plant protease inhibitors**	**Disease control and Clinical trial phase**	**Dose**	**References**
BBI	Cancer chemopreventive agent	800 chymotrypsin inhibitor units	Armstrong et al., 2000a
	Oral Leukoplakia: Phase-I		
	Oral Leukoplakia: Phase-IIa	200–1066 chymotrypsin inhibitory units	Armstrong et al., 2000b
	Phase-IIb	300 C.I. Units twice a day	Meyskens et al., 2010; Armstrong et al., 2013
BBI	Cancer preventive or anti-inflammatory agent: Phase-I	5 mg/mL	Malkowicz et al., 2003
BBI or BBIC	Multiple sclerosis Encephalomyelitis	1 mg/day of BBI or 3 mg/day of BBIC	Gran et al., 2006; Touil et al., 2008
BBIC	Prostatic hyperplasia,	100–800 chymotrypsin inhibitory units	Malkowicz et al., 2001; Kennedy and Wan, 2002; Kennedy, 2006
BBI	Ulcerative colitis		Lichtenstein et al., 2008

Until now, finished clinical trials did not show any toxicity or neutralizing antibodies against BBIC have been reported in patients. Armstrong et al. (2000a) reported the safety and nontoxic nature of BBI and BBIC in phase I trial of BBIC administered as an oral troche in patients with oral leukoplakia. The safety of BBIC has also been verified in other human clinical trials. The Phase-IIa trial of BBIC demonstrated simultaneous changes in neu protein levels and protease activity in patients treated with daily doses of 600 C.I.U of BBIC, administered as 300 C.I.U twice a day as 3 grams of BBIC (Armstrong et al., 2000b). This Phase-IIb clinical trial of BBIC did not show many significant differences between placebo and BBIC treatment. However, both BBIC treatment and placebo group showed statistically substantial decreases in total lesion area (Armstrong et al., 2013). Gran et al. (2006) found that BBIC administration to Lewis rats with experimental autoimmune encephalomyelitis (EAE) considerably suppressed the disease. Later, Touil et al. (2008) tested the purified BBI effects on clinical and histopathological responses of EAE in two models (relapsing/remitting EAE in SJL/J mice and chronic EAE in C57BL/6 mice). Treatment with BBI (1 mg/day of BBI or 3 mg/day of BBIC) in both EAE models significantly effected disease parameters such as onset, severity, weight loss, inflammation, and demyelination. Moreover, it significantly reduced the incidence of optic neuritis and prevented loss of retinal ganglion cells (Touil et al., 2008). Kennedy and Wan (2002) found that BBIC had a significant inhibitory effect on the growth and clonogenic survival of BRF-55T, 267B1/Ki-ras, LNCaP, and PC-3 cells. Dr. Kennedy's group and others carried out six human clinical trials for the following issues: cancer prevention on oral leukoplakia, treatment of benign prostatic hyperplasia, prostate cancer detection, and treatment, treatment of ulcerative colitis, gingivitis, or esophagitis (Malkowicz et al., 2001; Kennedy, 2006). A double-blind, randomized, phase I clinical trial was conducted in 19 males with Benign prostatic hyperplasia, which is an initial stage of prostate cancer and lower urinary tract symptoms (Malkowicz et al., 2001). This study demonstrated that BBIC treatment for 6 months reduced levels of prostate-specific antigen (PSA) which is a clinical marker for prostate cancer, and prostate volume in patients. Further clinical studies will be required to determine the potential of BBIC as prostate cancer chemopreventive agent. BBIC shows promise to become an effective nontoxic chemopreventive agent based on results of extensive preclinical studies, and Phase I and Phase IIa clinical trials. BBIC has dose-related clinical activity against oral leukoplakia and modulates levels of Neu (Neu immune histochemical staining intensity for lesions) and protease activity (Armstrong et al., 2003). Wan et al. (1999a) have reported a specific substrate hydrolysis methodology to measure the protease activity of human oral mucosal cells. Eventually authors have described the connection between neu oncogene expression and protease activity in patients that are registered for an oral cancer prevention trial using (BBIC) as the preventive agent. A completed clinical trial was performed to examine the safety and possible benefits of BBIC in patients with active ulcerative colitis (Lichtenstein et al., 2008). BBI demonstrated anti-inflammatory effects in patients with ulcerative colitis without toxicity. Investigation and identification of natural plant protease inhibitors and synthesis of peptidomimetic molecules have provided various beneficiary compounds that are successful in using among many human studies. Numerous plant protease inhibitors are undertaking an additional evaluation in human clinical trials.

## Conclusion

New research strategies are now focusing on the understanding of protease-regulated cascades, along with a specific selection of targets and improved inhibitor specificity. Many protease inhibitors have been found in natural sources and also have been synthesized for clinical uses. Among all, soybean BBI represent the most expansively studied members of the Bowman-Birk family, but related BBI from other dicotyledonous legumes (including *Cicer arietinum* (chickpea), *Phaseolus vulgaris* (common bean), *Lens culinaris* (lentil), and *Pisum sativum* (pea)) and from monocotyledonous grasses (*Poaceae*) (including (*Triticum aestivum*) wheat, *Oryza sativa* (rice) and *Hordeum vulgare* (barley) species), has been well recognized and characterized. Many pharmaceutical companies are taking keen interests in several plant protease inhibitors, which are currently in human trials (Clemente et al., 2010; Clemente and Arques, 2014). The sequences and crystal structure of many plant protease inhibitors are now available. But, still only a few are used in medicine and are in clinical trials (Majumdar, 2013). There are several advantages of using plant protease inhibitors compared to synthetic ones. Plant protease inhibitors can also be supplied through diet (e.g., rice, potato, legumes, soybean, corn, cucurbits, cereals etc.) by adding some extra plant based food preparations which will have no side effects on human body. Extensive research has to be done to find out the possible candidates of protease inhibitors that have therapeutic importance from plants. With the growing population and diseases, there is a need to explore more plant protease inhibitors useful in the treatment and control of human diseases. This review illustrates the enormous potential of the protease inhibitors from plant species in medicine.

## Author contributions

ZC initiated the project. SS contributed to the figures. SS, ZC wrote the manuscript.

## Funding

The authors thank the fund from Ministry of Education Singapore (RP 1/14 CZ).

### Conflict of interest statement

The authors declare that the research was conducted in the absence of any commercial or financial relationships that could be construed as a potential conflict of interest.
